# Editorial: Novel therapeutic approach in cancer: the role of ion channels and transporters

**DOI:** 10.3389/fphar.2024.1422127

**Published:** 2024-05-15

**Authors:** Concetta Altamura, Elena Lastraioli

**Affiliations:** ^1^ Department of Precision and Regenerative Medicine, Section of Pharmacology, School of Medicine, University of Bari Aldo Moro, Bari, Italy; ^2^ Department of Experimental and Clinical Medicine, University of Florence, Florence, Italy

**Keywords:** ion channel, cancer, transporters, pharmacology, cancer biology

In the last two decades, the study of ion channels and transporters in cancer has been exponentially growing. Many of these proteins have been implicated in several cellular processes as cancer cell proliferation, migration, invasion, tumor vascularization and metastasis, revealing them as key factors in cancer biology. Such view is in line with the association of cancer with the term oncochannelopathy.

We are happy to present a Research Topic of articles in Frontiers in Pharmacology highlighting ion channels and membrane transporters as new potential targets for cancer treatment.

The total Research Topic brings together unique contributions from original research to review articles covering the dysregulation of a variety of channels and transporters in multiple cancers, emphasizing the importance of these proteins for diagnostic, prognostic and therapeutic purposes.

Among ion channels, voltage-gated proton channels (Hv1) have been reported to play an important role in the pathogenesis and progression of several tumors, including glioma, colorectal and breast cancer. These proteins mediate proton extrusion for maintaining a balanced cytosolic pH in health and cancer cells. The review by Alvear-Arias et al. highlighted a strong relationship between the overexpression of Hv1 channel, tumor microenvironment acidification and cancer development, proposing this protein as a marker for malignancy and a promising therapeutic target to counter the development of solid tumors.

A peculiar role of Hv1 channels in immune system was described in the article by Peña-Pichicoi et al. These proteins are proposed as potential therapeutic targets to alleviate dysregulated immunosuppression in cancer because of their contribution in the immunosuppressive activity of myeloid-derived suppressor cells (MDSCs). However, few information is published regarding the Hv1 isoform diversity in mice and MCSCs. The authors identified six variants of Hv1 channels (Hv1.1-1.6) in mice and MDSCs and functionally characterized the different variants by macro-patch voltage clamp. The six variants presented varying gating properties.

Lavoro et al. presented an *in silico* study aimed at evaluating Solute Carrier (SLC) transporters in different tumors and healthy tissues. Moreover, the relevance of DNA methylation as an epigenetic mechanism of gene expression regulation was also investigated. The authors identified 62 differentially expressed SLC genes: two of them (*SLC4A4* and *SLC7A11*) are associated with outcome; *SLC24A5* and *SLC45A2* are positively correlated with anti-MEK and anti-RAF sensitivity. The expression of the SLCs was associated with methylation patterns of the promoter as well as of the gene body.

The review by Herrera-Quiterio and Encarnaciòn-Guevara provides a complete picture of Transmembrane proteins (TMEM) and their relevance in cell processes. Interestingly, TMEMs were evaluated in human cancer through transcriptomic and proteomic approaches and from such analyses it emerged that they are involved in all the key features of cancer cells such as migration, cell proliferation, invasion, epithelial-mesenchymal transition, intra-/extravasation, metastasis and response to antineoplastic drugs. The last association is of particular interest since some TMEMs are involved in chemoresistance therefore they might represent a candidate biomarker for prognosis and response to therapy.

Capitani et al. reviewed the role of ion channels in non-small and small cell lung cancer, including potassium, sodium, calcium, chloride, anion channels and nicotinic acetylcholine receptors. A variety of these proteins are remarkably expressed in both tumors, where they are involved in the development of various malignant behaviors, including cell proliferation, migration, invasiveness, and drug resistance. For some of these channels, a correlation of their expression levels with prognosis or resistance to chemotherapeutics has also been reported, supporting a putative role in predicting clinical outcome and defining cancer therapy. In line with this, the use of ion channel-targeting drugs, some of them already tested in clinical trials for other malignancies, may bring several benefits, thus opposing tumor development and resistance.

Therefore, the study of ion channels in cancer has become an interesting scientific field, attracting more exciting studies. We hope that this Research Topic will provide new insights into the current understanding of ion channels and transporters involved in cancers, encouraging the development of innovative ion channel-centered, therapeutic strategy for this disease.

